# Detailed Molecular Epidemiologic Characterization of HIV-1 Infection in Bulgaria Reveals Broad Diversity and Evolving Phylodynamics

**DOI:** 10.1371/journal.pone.0059666

**Published:** 2013-03-19

**Authors:** Ivailo Alexiev Ivanov, Danail Beshkov, Anupama Shankar, Debra L. Hanson, Dimitrios Paraskevis, Viara Georgieva, Lyudmila Karamacheva, Hristo Taskov, Tonka Varleva, Ivaylo Elenkov, Mariana Stoicheva, Daniela Nikolova, William M. Switzer

**Affiliations:** 1 National Reference Laboratory of HIV, National Center of Infectious and Parasitic Diseases, Sofia, Bulgaria; 2 Division of HIV/AIDS Prevention, National Center for HIV/AIDS, Viral Hepatitis, STD, and TB Prevention, Centers for Disease Control and Prevention, Atlanta, Georgia, United States of America; 3 National Retrovirus Reference Center, Department of Hygiene Epidemiology and Medical Statistics, Medical School, University of Athens, Athens, Greece; 4 Program “Prevention and Control of HIV/AIDS”, Ministry of Health, Sofia, Bulgaria; 5 Hospital for Infectious and Parasitic Diseases, Sofia, Bulgaria; 6 Medical University, Plovdiv, Bulgaria; 7 Medical University, Clinic of Infectious Diseases, Varna, Bulgaria; Institut Pasteur of Shanghai, Chinese Academy of Sciences, China

## Abstract

Limited information is available to describe the molecular epidemiology of HIV-1 in Bulgaria. To better understand the genetic diversity and the epidemiologic dynamics of HIV-1 we analyzed 125 new polymerase (*pol*) sequences from Bulgarians diagnosed through 2009 and 77 *pol* sequences available from our previous study from persons infected prior to 2007. Epidemiologic and demographic information was obtained from each participant and phylogenetic analysis was used to infer HIV-1 evolutionary histories. 120 (59.5%) persons were infected with one of five different HIV-1 subtypes (A1, B, C, F1 and H) and 63 (31.2%) persons were infected with one of six different circulating recombinant forms (CRFs; 01_AE, 02_AG, 04_cpx, 05_DF, 14_BG, and 36_cpx). We also for the first time identified infection with two different clusters of unique A-like and F-like sub-subtype variants in 12 persons (5.9%) and seven unique recombinant forms (3.5%), including a novel J/C recombinant. While subtype B was the major genotype identified and was more prevalent in MSM and increased between 2000–2005, most non-B subtypes were present in persons ≥45 years old. CRF01_AE was the most common non-B subtype and was higher in women and IDUs relative to other risk groups combined. Our results show that HIV-1 infection in Bulgaria reflects the shifting distribution of genotypes coincident with the changing epidemiology of the HIV-1 epidemic among different risk groups. Our data support increased public health interventions targeting IDUs and MSM. Furthermore, the substantial and increasing HIV-1 genetic heterogeneity, combined with fluctuating infection dynamics, highlights the importance of sustained and expanded surveillance to prevent and control HIV-1 infection in Bulgaria.

## Introduction

The rapidly evolving human immunodeficiency virus type 1 (HIV-1) is characterized by enormous genetic heterogeneity and is divided phylogenetically into four major groups: M (major), N (new), O (outlier), and the recently identified group P from a cross-species transmission from an SIV-infected gorilla [1]. Nine subtypes of HIV-1 group M (A–D, F–H, J and K) and few sub-subtypes, e.g. F1, F2, and A1 – A4 are currently recognized. In addition, a great variety of circulating recombinant forms (CRFs) and unique recombinant forms (URFs) have been identified adding to the growing genetic complexity of HIV-1 [2]. The unequal worldwide distribution of the different HIV-1 genotypes results from the global transmission and spread of certain variants or the limited spread of local endemic strains [2]. Subtype B is predominant in the Americas, Western Europe, and Australia [3], [4], subtype A prevails in Russia and the former Soviet Union (FSU) countries, and is also prevalent in Africa. Subtype C is the most abundant genetic form in South and Eastern Africa, South East Asia, and worldwide followed by subtypes A and B. CRFs and URFs are widely distributed in countries where different subtypes co-circulate [5], [6]. In Eastern Europe, Russia, Ukraine, Belarus and Moldova the HIV-1 epidemic is dominated by subtype A, followed by subtype B and CRF03_AB [7]–[10]. In the Baltic countries, subtype B predominates in Lithuania, subtype A1 is more common in Latvia, and the rare genetic form CRF06_cpx prevails in Estonia [11]–[13].

The HIV-1 epidemic in the Balkan region has been affected by various historic and socio-economic factors. Recently, several studies of HIV-1 molecular epidemiology in this region have reported a wide variety of introduced and prevalent HIV-1 genotypes with specific subtypes predominating in each country. Like Western Europe, subtype B is also the primary subtype here, but to a lesser extent in Croatia, Greece, Montenegro, Slovenia, Serbia and Turkey. In contrast, the principal subtype in Albania is A1, 95% of HIV-1 in Romania is subtype F1, and various subtypes and CRFs have been found in Cyprus [14]–[24]. In addition, a study in Greece identified outbreaks within specific risk groups after the introduction of the A1 genotype [25]. Thus, the genetic diversity of HIV-1 in the Balkan region is much more complex compared to that reported in Western Europe.

Current epidemiological data indicate that the impact of the HIV-1 epidemic in Bulgaria, as part of the Central European region, is still limited [26]. From the beginning of 1986 to the end of 2009, which is the period of the current study, 1,100 HIV/AIDS cases were registered, including 327 persons with AIDS, 798 (72.6%) persons were male and 302 (27.5%) were female. Most of infections were attributed to heterosexual transmission 747 (67.9%), followed by intravenous drug use (IDU) 224 (20.4%), from men who have sex with men (MSM) 104 (9.5%), blood transfusion 17 (1.6%), and mother-to-child transmission 8 (0.7%). Early in the epidemic most infections were acquired by heterosexual contact, while more recently HIV-1 infection was more common in IDUs (43.3%), followed by MSM (15.2%). The annual number of HIV-1 diagnoses increased from 15–20 in the early 1990s to 171 in 2009.

Given the observed recent changes in the HIV-1 epidemic in Bulgaria with regard to rising proportions of infected IDUs and MSMs, and the specific geographic location of Bulgaria at the intersection of Western Europe, Eastern Europe and the Middle East, defining the diversity of HIV genotypes in Bulgaria in different vulnerable populations is epidemiologically important [27]–[29]. Thus, the objective of the present study was to perform a comprehensive molecular epidemiological survey of HIV-1 diversity in Bulgaria and to investigate the prevalence of viral variants circulating among different risk groups to better characterize the HIV-1 epidemic. Polymerase (*pol*) sequences were PCR-amplified from fresh blood samples collected from 125 newly identified HIV-1-infected persons representative of different risk groups from different Bulgarian areas and from different years of the epidemic. Detailed phylogenetic analyses of these new HIV-1 sequences, combined with 77 HIV-1 sequences previously reported from our group from persons infected during 1986–2006 and the appropriate reference sequences, helped to better define the molecular epidemiology of HIV-1 infection in Bulgaria.

## Materials and Methods

### Ethics statement

All patients provided written informed consent to participate in this study. Our study received approval by the Ethical Committee at the National Centre of Infectious and Parasitic Diseases, Sofia Bulgaria. NCIPD IRB IORG0003505.

### Study population and specimen preparation

In addition to 77 patients from our previous study in 2007 [27], we collected blood samples from 125 new persons for a total of 202 (18.36%) patients from the 1,100 persons diagnosed with HIV-1 infection in Bulgaria from the beginning of the epidemic until 2009. Convenience specimens were collected from different vulnerable populations, including MSM, IDUs, offspring of infected mothers, and blood product recipients. The blood samples were collected at the National HIV Confirmatory Laboratory and in clinics in the capital city of Sofia and the other two main cities of the country, Plovdiv and Varna. All patients consented to the testing and data collection procedures. Following specimen collection and patient interviews to obtain demographic and clinical information, the blood samples were linked to the demographic and clinical data through an anonymous numerical code in accordance with the ethical standards of Bulgaria. Plasma was obtained from the blood specimens by centrifugation at the National HIV Confirmatory Laboratory in Sofia and stored at 80°C as previously described [27].

### Polymerase (*pol*) gene RT-PCR and sequence analysis

Viral RNA was extracted from one ml plasma using the QIAamp® Ultra Sens™ Virus Kit 50 (QIAGEN, Cat. No.53704). Generation of the protease (PR) and reverse transcriptase (RT) sequences of the HIV-1 *pol* gene was performed using either the Viroseq HIV-1 Genotyping Test (Abbott) and/or TruGene DNA Sequencing System (Siemens Healthcare) RT-PCR kits at the National HIV Confirmatory Laboratory in Sofia, Bulgaria. Automated DNA sequencing was done using an Applied Biosystems model 310 sequencer or an OpenGene DNA sequencing system (Visible Genetics, Siemens) following the manufacturer's protocol.

All 125 new HIV-1 *pol* sequences were first analyzed using the internet-based REGA HIV-1&2 Automated Subtyping Tool (Version 2.0) (http://jose.med.kuleuven.be/genotypetool/html/subtypinghiv.html) [30], the COntext-based Modeling for Expeditious Typing, version 0.2 (COMET HIV-1/2) – http://comet.retrovirology.lu/, and the Geno2pheno v3.3 (www.geno2pheno.org/) programs to obtain preliminary HIV-1 subtype classification. Possible genetic composition of URF *pol* sequences was inferred using the BLAST sliding window algorithm implemented at NCBI (www.ncbi.nlm.nih.gov/projects/genotyping) and used to guide selection of reference sequences in bootscanning analysis. Manual bootscan analysis was done with the program SimPlot to confirm selected subtypes using the F84 nucleotide substitution model and a sliding window of 200-bp, a 40-bp step, with the transition/transversion ratio determined empirically [31]. Percent nucleotide identities and distances were calculated using alignments of selected sequences using the software Geneious Pro v5.5.5 and MEGA5 using TN93+G+I as a substitution model, respectively. Detection of recombination was also evaluated using the programs RDP, 3Seq, GENECON, MaxChi, and Chimaera, implemented in RDP v3 [32].

Individual BLAST searches of all 125 new sequences was performed and the most similar GenBank sequences for each subtype and CRF were downloaded from the HIV Los Alamos sequence database for further sequence analysis (http://www.hiv.lanl.gov/). To obtain a comprehensive description of the molecular epidemiology of HIV-1 in Bulgaria we also included in the analyses 77 HIV-1 sequences from Bulgaria previously reported by our group. Nucleotide alignments were prepared using Clustal W v1.6 in the MEGA5 and BioEdit software packages followed by manual editing [33], [34]. Several alignments were made for phylogenetic analysis, including the complete data set consisting of 125 new and 77 previously reported Bulgarian sequences, phylogenetically similar sequences from the BLAST search and HIV-1 subtype reference sequences from 2011 available at the Los Alamos HIV database. To investigate further the molecular epidemiology of HIV-1 in Bulgaria, we also analyzed subsets of Bulgarian and reference sequences by using selected genotype clusters inferred by maximum likelihood (ML) Bayesian analysis.

The best fitting distance model of nucleotide substitution for each alignment was inferred using the ML method with goodness of fit measured by the Bayesian information criterion in MEGA5. The best fitting nucleotide substitution model for the phylogenetic alignments was inferred to be the general time reversible model (GTR) with discrete gamma and invariant among-site rate variation. Phylogenetic relationships were inferred using the ML method in MEGA5 and using Bayesian analysis with BEAST v1.6.2 [35] program. Stability of the ML tree topologies was tested using 1,000 nonparametric bootstrap replicates, whereas statistical support for the inferred Bayesian trees was assessed by posterior probabilities. For the Bayesian analyses, a relaxed molecular clock model was used and each run consisted of two independent 800×10^6^ Markov chain Monte Carlo (MCMC) generations with sampling every 800,000^th^ generation and a constant coalescent tree prior. Convergence of the MCMC was assessed by calculating the effective sampling size (ESS) of the runs using the program Tracer v1.5 (http://beast.bio.ed.ac.uk/Tracer). All parameter estimates showed significant ESSs >1,200. The tree with the maximum product of the posterior clade probabilities (maximum clade credibility tree) was chosen from the posterior distribution of 8,001 sampled trees after burning in the first 1,000 sampled trees with the program TreeAnnotator version 1.6.2 [36].

Potential epidemiologic clusters were defined using a stringent set of criteria and included those sequences grouping together in phylogenetic analysis with posterior probabilities ≥0.97, ≥96% ML bootstrap support, and sharing >90% nucleotide identity per total sampling period between related sequences. The latter estimate is based on the 10^−3^ substitution rate for HIV-1 *pol* sequences generating a divergence rate of 1–2%/year between founder and transmitted viruses [36]. For example, HIV sequences sampled 5 years after a transmission event would expect to be 5–10% divergent. Antiretroviral resistance-associated mutations were detected using the Stanford genotypic resistance algorithm (http://sierra2.stanford.edu) and stripped from partial alignments containing potential transmission clusters to minimize clustering artifacts.

### Accession numbers

GenBank accession numbers for the 125 new *pol* sequences are JQ259060–JQ259184.

### Statistical analysis

To assess the epidemiological associations with each of the major HIV-1 subtype groups, multivariable logistic regression models were constructed, controlling for the following demographic and risk mode variables: age at diagnosis, gender, transmission mode, infection abroad, and diagnosis period. Each subtype group (B, other major subtypes combined (C, A1, F1, H), 01_AE, 02_AG, rare CRFs, and URFs) was modeled relative to all other subtype groups collectively. Odds ratios and 95% confidence limits were calculated. Single variable logistic regression models were also performed and results are reported for findings that differed from those of the multivariable models. In addition, linear trends (degree of freedom  = 1) in subtype prevalence and in proportion of infections by risk transmission category were assessed using the Cochran-Armitage trend test. Differences in subtype diversity by population subgroups were assessed using a Fisher-Freeman-Halton test for nominal independence.

## Results

### Study population demographics

141 (69.8%) of 202 persons were male and 61 (30.2%) were female representative of the total HIV-1-infected population (Table 1). The majority of persons (140 (69.3%)) attributed their infection to heterosexual contact, 34 (16.8%) were MSM, 19 (9.4%) were IDUs, 5 (2.5%) persons were infected by blood transfusion and 4 (2%) newborns were infected by vertical transmission (Table 1). The youngest individual in the study population was a newborn, the oldest was 62 years old, and the mean age of the population studied was 31.8 years. According to patient data, 175 (86.6%) persons were presumed to be infected in Bulgaria while 27 (13.4%) reported possibly acquiring infection abroad while traveling or living in Europe (England (n = 1), Greece (n = 3), Cyprus (n = 1), Germany (n = 2), Italy (n = 1), Romania (n = 1), Spain (n = 2), Russia (n = 1), Africa (Nigeria (n = 2), Democratic Republic of Congo (n = 1), Ghana (n = 1), Libya (n = 1), South Africa (n = 2), or Nicaragua (n = 1). For seven persons the purported country where infection occurred was not specified.

**Table 1:** Distribution of age, gender, route of infection, place of presumed infection, and year of diagnosis in Bulgaria.

	Study Population	Registered individuals with HIV-1 in Bulgaria till 2009
	Number (%)	Number (%)
**Total**	202 (100)	1100 (100)
**Age (years)**		
<20	14 (6.9)	73 (6.6)
20–44	163 (80.7)	887 (80.6)
≥45	25 (12.4)	140 (12.7)
**Gender**		
Male	141 (69.8)	798 (72.6)
Female	61 (30.2)	302 (27.5)
**Route of infection**		
Heterosexual	140 (69.3)	747 (67.9)
MSM	34 (16.8)	104 (9.5)
Hemotransfusion	5 (2.5)	17 (1.6)
IDUs	19 (9.4)	224 (20.4)
Vertical	4 (2.0)	8 (0.7)
**Presumed country of infection**		
Bulgaria	175 (86.6)	981 (89.2)
Abroad	27 (13.4)	119 (10.8)
**Diagnosis period**		
1986–1995	22 (10.9)	146 (13.3)
1996–1999	25 (12.4)	110 (10)
2000–2005	78 (38.6)	324 (29.5)
2006–2009	77 (38.1)	520 (47.3)

### High diversity of HIV-1 circulating in Bulgaria

**Figure 1:**
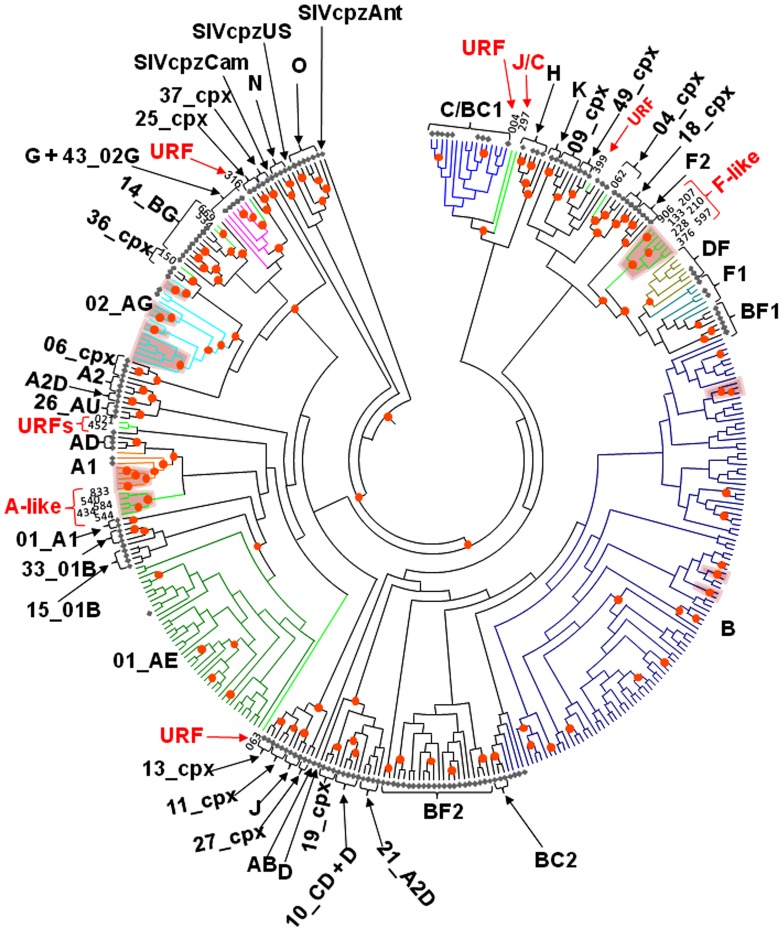
Inferred genetic relationships of 202 HIV-1 polymerase (*pol*) sequences from persons in Bulgaria. Bayesian inference performed using the BEAST software package and a relaxed molecular clock and constant population size tree prior. 170 HIV-1 reference *pol* sequences were used for genetic comparison. Reference sequences are indicated with a grey diamond. The maximum clade credibility tree is shown. Final alignment length is 804 nucleotides. Posterior probabilities greater than 0.97 at nodes are shown with orange dots. Clades shaded with rose-colored trapezoids indicate possible epidemiologically-linked sequences supported with posterior probabilities ≥0.97, maximum likelihood bootstrap support ≥97%, and having <10% divergence per sampling period. Genotype classification of the Bulgarian sequences is shown with respect to the reference sequences. URFs and rare CRF lineages in Bulgaria are shown with bright green branches. Branches of major subtypes and CRF are highlighted with random colors. All other branches are black.

**Figure 2:**
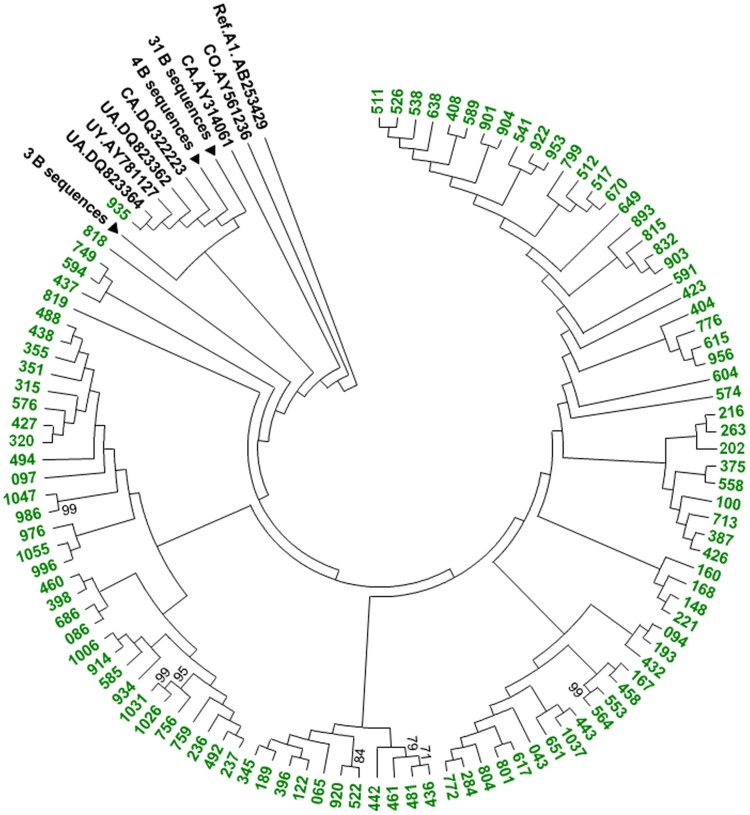
Phylogenetic relationship of Bulgarian HIV-1 subtype B polymerase sequences. The 691-bp alignment was composed of 104 Bulgarian HIV-1 B strains and 45 subtype B reference sequences from the Los Alamos HIV database. Antiretroviral resistance-associated mutations were stripped from the alignment. The tree was rooted by using an HIV-1 subtype A1 strain as the outgroup. Tree topology was inferred using maximum likelihood analysis implemented in MEGA5. Support for each node was determined using 1,000 bootstrap replications with only values ≥70 shown. Scale bar indicates the number of nucleotide substitutions per site. Nearly identical tree topologies were also obtained with Bayesian analysis. Bulgarian sequences are shown using green branches and taxon names.

**Figure 3:**
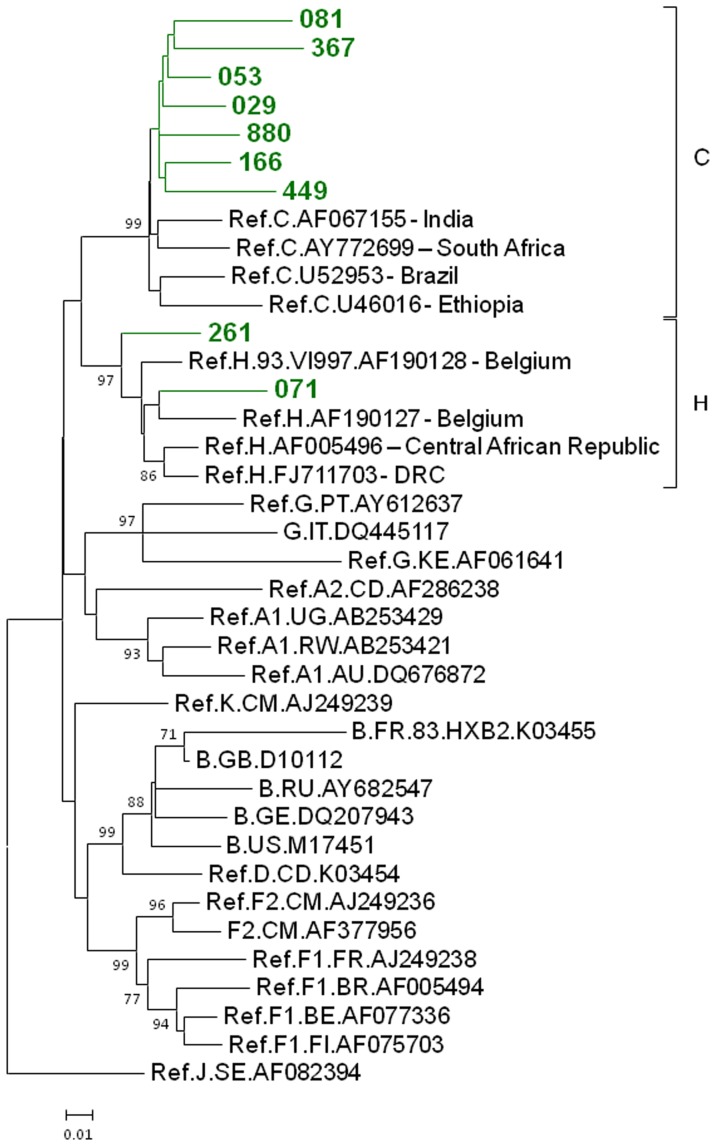
Inferred phylogenetic relationships of Bulgarian HIV-1 subtypes C and H. Tree structure was inferred using maximum likelihood analysis of polymerase sequences implemented in MEGA5. Support for each node was determined using 1,000 bootstrap replications with only values ≥70 shown. Scale bar indicates the number of nucleotide substitutions per site. Antiretroviral resistance-associated mutations were stripped from the alignments. Nearly identical tree topologies were also obtained with Bayesian analysis. The 690-bp alignment consisted of 9 HIV-1 C and H strains from Bulgaria and 29 Group M reference sequences from the Los Alamos HIV database. The tree was rooted by using an HIV-1 subtype J strain as the outgroup. Bulgarian sequences are shown using green branches and taxon names.

**Figure 4:**
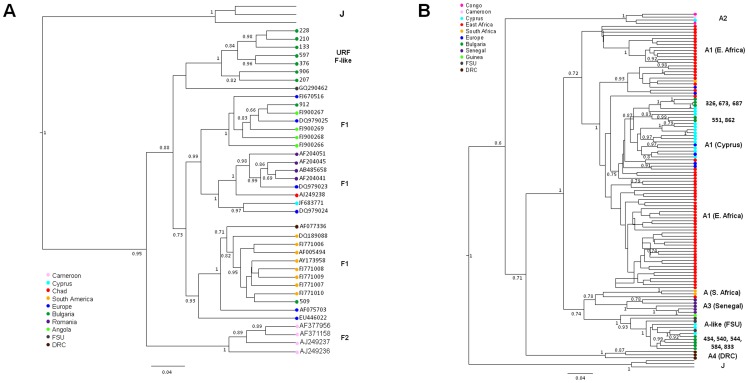
Identification of novel HIV-1 in Bulgaria. Phylogenetic relationships were inferred using Bayesian methods implemented in the program BEAST using a relaxed molecular clock and constant population size tree prior. Antiretroviral resistance-associated mutations were stripped from the alignment. Posterior probabilities >0.6 are provided at the nodes. The scale bar indicates relative units of time. Nearly identical tree topologies were also obtained with maximum likelihood analysis. (**A**) **F-like sub-subtypes.** The 758-bp alignment was composed of 9 subtype F and F-like sub-subtype polymerase sequences from Bulgaria and 43 subtype F reference sequences from the Los Alamos HIV database and highly related sequences identified using BLAST. The tree was rooted by using three HIV-1 subtype J strains as the outgroup. (**B**) **A-like sub-subtypes.** The 754-bp alignment consisted of 10 subtype A and A-like sub-subtype polymerase sequences from Bulgaria and 121 subtype A reference sequences from the Los Alamos HIV database and highly related sequences identified using BLAST. Antiretroviral resistance-associated mutations were stripped from the alignment. The tree was rooted by using three HIV-1 J strains as the outgroup.

**Figure 5:**
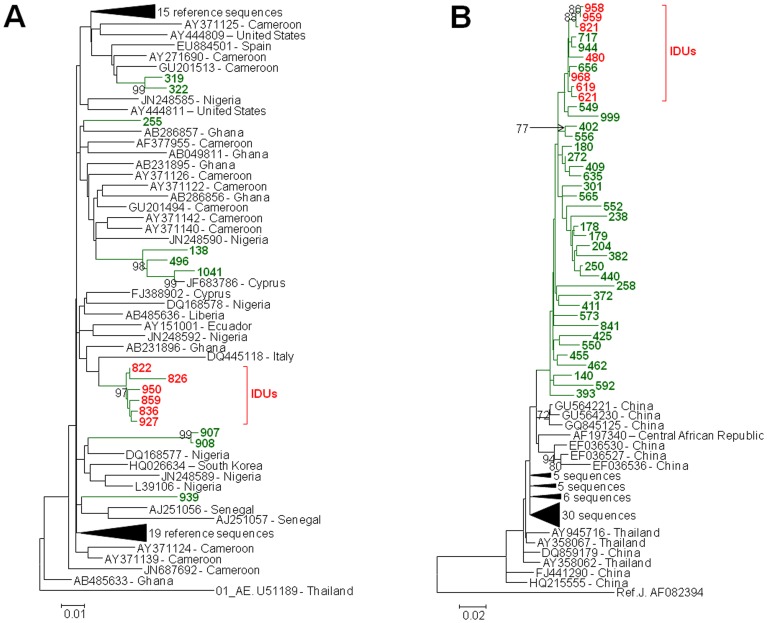
Inferred phylogenetic relationships of Bulgarian HIV-1 subtypes. Tree structure was inferred using maximum likelihood analysis of polymerase sequences implemented in MEGA5. Support for each node was determined using 1,000 bootstrap replications with only values ≥70 shown. Scale bar indicates the number of nucleotide substitutions per site. Antiretroviral resistance-associated mutations were stripped from the alignments. Nearly identical tree topologies were also obtained with Bayesian analysis. (**A**) **Subtype 02_AG.** The 777-bp alignment was composed of 15 HIV-1 02_AG strains from Bulgaria and 71 02_AG reference sequences from the Los Alamos HIV database. The tree was rooted by using HIV-1 01_AE strain as the outgroup. Bulgarian sequences are shown using green branches and taxon names. Taxon names in red represent Bulgarian IDUs. (**B**) **Circulating recombinant form (CRF) 01_AE.** The 689-bp alignment was composed of 40 HIV-1 CRF 01_AE strains from Bulgaria and 60 subtype CRF 01_AE reference sequences from the Los Alamos HIV database. The tree was rooted by using HIV-1 subtype J as the outgroup. Bulgarian sequences are shown using green branches and taxon names. Taxon names in red represent Bulgarian IDUs.

**Figure 6:**
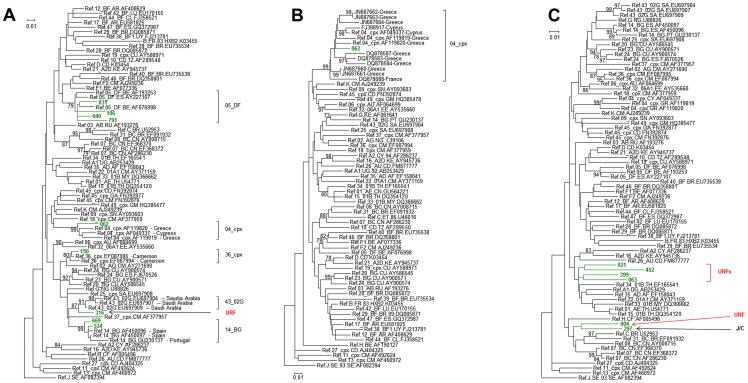
Inferred phylogenetic relationships of Bulgarian HIV-1 subtypes. Tree structure was inferred using maximum likelihood analysis of polymerase sequences implemented in MEGA5. Support for each node was determined using 1,000 bootstrap replications with only values ≥70 shown. Scale bar indicates the number of nucleotide substitutions per site. Antiretroviral resistance-associated mutations were stripped from the alignments. Nearly identical tree topologies were also obtained with Bayesian analysis. (**A**) **Multiple Circulating and unique recombinant forms (CRF and URF, respectively).** The 696-bp alignment was composed of one CRF04_cpx, four CRF05_DF, two CRF14_BG, and one CRF36_cpx strains and one CRF43_02G-like strain from Bulgaria and 71 related reference sequences from the Los Alamos HIV database. The tree was rooted by using an HIV-1 subtype J strain as the outgroup. Bulgarian sequences are shown using green branches and taxon names. (**B**) **CRF04_cpx.** The 1043-bp alignment was composed of one HIV-1 CRF04_cpx1 strain from Bulgaria and 69 reference sequences from the Los Alamos HIV database and related sequences identified by BLAST. The tree was rooted by using an HIV-1 subtype J sequence as the outgroup. Bulgarian sequence is depicted using a green branch and taxon name. **(C) Identification of novel HIV-1 URFs in Bulgaria.** The 696-bp alignment was composed of six HIV-1 URF strains from Bulgaria and 68 reference sequences from the Los Alamos HIV database. The tree was rooted by using an HIV-1 subtype J strain as the outgroup. Bulgarian sequences are shown using green branches and taxon names. Bulgarian sequence #297 was shown by SimPlot analysis to be a novel recombinant of subtype J and C sequences.

**Figure 7:**
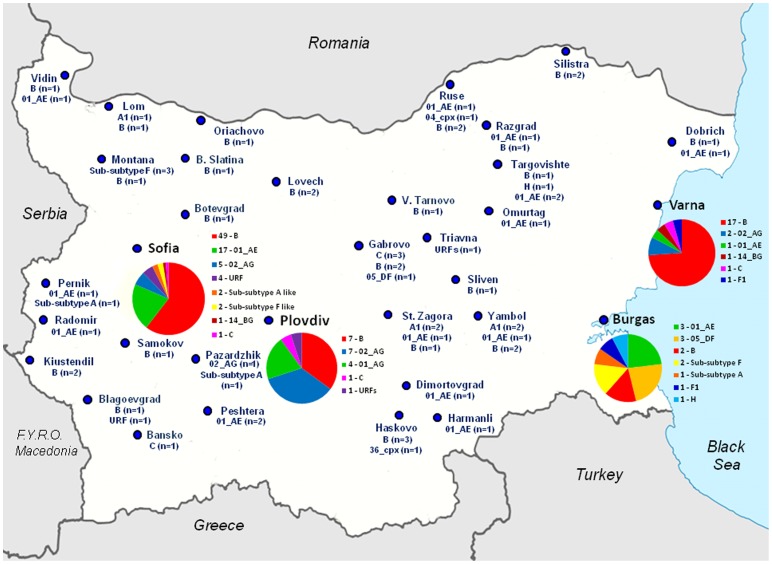
Geographic distribution of HIV-1 subtypes within Bulgaria. Pie charts show genotype distribution within the most populous cities of Sofia, Plovdiv, Burgas, and Varna. Numbers of each subtype are in parentheses for the smaller towns.

Phylogenetic analysis using ML and Bayesian MCC methods of *pol* sequences classified 120 (59.5%) of the 202 Bulgarian HIV-1 samples to five different major subtypes (Figs. 1, 2, 3, 4, 5, 6, 7, Table 2). 104 (51.5%) were subtype B (Figs. 1 and 2), 7 (3.5%) subtype C, and 2 (1.0%) were subtype H (Fig. 3). 5 (2.5%) subtype A1, and 2 (1.0%) subtype F1 (Fig. 4). In addition, 82 (40.6%) sequences were classified in at least ten different CRFs or URFs: 40 (19.8%) CRF01_AE, and 15 (7.4%) CRF02_AG (Fig. 5), 4 (2%) CRF05_DF, 2 (1.0%) CRF14_BG, one (0.5%) CRF04_cpx, and one (0.5%) CRF36_cpx (Fig. 6), and 19 (9.4%) sequences were found to be URFs (n = 7) or possible sub-subtype variants (n = 12) (Figs. 1, 4 and 6) (Table 2).

**Table 2:** HIV-1 subtype diversity in samples from 202 Bulgarian residents.

Subtype	Number	Prevalence (95% confidence limits)	Travel Outside of Bulgaria (number of persons)^1^
B	104	51.5 (44.4, 58.6)	England (1), Greece (1), Italy (1), South Africa (1), Romania (1), Spain (1), Germany (2), unspecified (1)
C	7	3.5 (1.4, 7.0)	unspecified (1)
A1	5	2.5 (0.8, 5.7)	Greece (1)
F1	2	1.0 (0.1, 3.5)	(0)
H	2	1.0 (0.1, 3.5)	(0)
01_AE	40	19.8 (14.5, 26.0)	Nicaragua (1)
02_AG	15	7.4 (4.2, 12.0)	Ghana (1), Cyprus (1), unspecified (1)
04_cpx	1	0.5 (0.0, 2.7)	(0)
05_DF	4	2.0 (0.5, 5.0)	Democratic Republic of Congo (1), unspecified (1)
14_BG	2	1.0 (0.1, 3.5)	Spain (1)
36_cpx	1	0.5 (0.0, 2.7)	Libya (1)
URFs and new sub-subtypes	19	9.4 (5.8, 14.3)	Greece (1), Russia (1), South Africa (1), Nigeria (2), unspecified (3)

1. Self-reported travel outside of Bulgaria which may not accurately reflect geographic origin of infection.

The location of the various genotypes was plotted on a map of Bulgaria to examine trends in their geographic distribution (Figure 7). The majority of infections and greatest heterogeneity was observed in the capital of Sofia, followed by the three other major cities of Plovdiv, Burgas, and Varna. The most common subtypes B and CRF01_AE showed the widest distribution across Bulgaria. The majority of the URFs were located in and around Sofia.

### Identification of HIV-1 clusters within Bulgaria

Using stringent phylogenetic and nucleotide identity criteria as evidence of possible epidemiologically linked HIV-1 sequences, we identified 10 clusters of highly associated sequences (Figure 1). The majority of the clusters occurred in the CRF02_AG and subtype B clades (Figure 1). Within CRF02_AG there was one group of six, and three pairs of related sequences (Figures 1 and 5A). A monophyletic lineage with high posterior probability containing six sequences (patients 927, 859, 822, 836, 826,950 and 836) was obtained from specimens collected over about a year from male IDUs who all lived in Plovdiv and the neighboring city of Haskovo. All six sequences shared >97.4% nucleotide identity. A heterosexual pair (907 and 908) from Sofia reported contact with each other and was infected with subtype CRF02_AG and shared 99.4% nucleotide identity (Figures 1 and 5A). Heterosexual spouses (319 and 322) were both infected with subtype CRF02_AG *pol* sequences that clustered together with a 1.0 posterior probability, 99% bootstrap support, and which shared 98% nucleotide identity (Figures 1 and 5A). The fourth cluster contained sequences from three heterosexual females (138, 496, 1041) of which one (138) reported possible infection while in Ghana [37]. HIV-1 CRF02_AG in person 1041 was also highly related to that from an individual from Cyprus (Figures 1 and 5A). All four sequences in this cluster shared >95% nucleotide identity.

Seven sequences (133, 207, 210, 228, 376, 597, and 906) were unique and clustered together with strong support as sister clades of subtypes CRF05_DF, F1 and BF(Figures 1 and 4A). Sequence analysis showed that this F-like cluster shared 90.7–97.1% nucleotide identity which is greater than that within F1 (94–96.5%), F2 (95.8–97.1%), and CRF05_DF (94.9–96.6%) in this region of *pol*. Likewise, the F-like Bulgarian sequences were equidistant from the F1, F2, and CRF05_DF subtypes sharing 75–79% nucleotide identity which is greater than the range of sequence identity between F1, F2, and CRF-05_DF in this region (89–96%). Bootscan analysis showed the F-like sequences to be mostly composed of F1 sequences but with weak support suggesting they contain unidentified sequences (Figure S1A–G). Analysis of F2 sequences has also determined that they may contain fragments of an uncertain origin [38]. Additional phylogenetic analysis including sequences from BLAST analysis showed these seven sequences clustered with strong support with an F-variant from the former Soviet Union (FSU) (Figure 4A) [39]. Thus, we are tentatively classifying these new Bulgarian sequences as F-like sub-subtype variants.

Similarly, five persons (434, 540, 544, 584, and 833) were infected with another novel sub-subtype that formed a distinct lineage between A1 and CRF01_AE in the phylogenetic analysis (Figures 1 and 4B). Bootscan analysis showed the genetic structure of these five sequences was similar and is composed of mostly A1 and unidentified sequences (Figure S2A–E). The nucleotide identities within this group of five sequences (94–96.9%) was similar to that seen within A1 (94.2–95.3%), A2 (93.8–95.3%), A3 (93.8–98.6%), A4 (94.1–94.9%), and CRF01_AE (95.3–97.5%). In addition, this group of five Bulgarian sequences and the A1, A2, A3, A4, CRF01_AE reference sequences were nearly equidistant from each other sharing about 90–95%, which is similar to that between subtypes B and D in this region. The distinct relationship of these five A-like sequences to other subtype A sequences was also supported by phylogenetic analysis as were the A1, A2, A3, and A4 sub-subtypes (Figure 4B). The five A-like sequences clustered with other *pol* sequences from HIV-1 infected persons from Africa, FSU, and persons from Cyprus who reported infection in FSU that have also been described as A variants or A sub-subtypes and were shown to originate from Africa [40]. These findings are consistent with our previous results showing an A clade from FSU but for which we classified these variants as A1 genotypes [27]. Interestingly, three of these five persons reported acquiring infection abroad; one in Russia (833), one in Greece (544), and one (540) who did not define a country and is consistent with the prevalence of these A-variants in FSU and Greece. Recombination was not detected in any of the seven F-like or five A-like *pol* sequences using the program RDP and the phylogenetic results were not influenced by the presence of antiretroviral drug resistant mutations (data not shown). Combined, and in accordance with the 1999 HIV-1 nomenclature proposal [41], these data suggest these Bulgarian A- and F-like sequences may represent sub-subtypes first reported in FSU but originating from Africa [40].

The remaining different but highly related sequence sets were composed of mostly subtype B infections (three sets) and one group of five subtype A1 sequences (326, 673, 687, 551 and 862) (Figures 1 and 4B). Male participants 326, 673, and 687 were all infected with HIV subtype A1 that shared >96.5% nucleotide identity over the 5 to 6 years of sampling and clustered together with high statistical support (Figures 1 and 4B). Sequences from persons 326 and 673 were 100% identical while those from persons 326 and 687 were 96.5% identical. Persons 673 and 687 were both MSMs, while person 326 reported being heterosexual. Person 673 lived in Greece and like person 687 was MSM. Person 326 reported being heterosexual. Patients 551 and 862 were male and female heterosexuals, respectively. Although contact information was not available for this pair, the high HIV *pol* sequence nucleotide identity (97%) and strong phylogenetic support suggests a significant shared evolutionary history of their infection most likely with persons from Cyprus (Figure 4B). Two MSM groups from Sofia (756, 1026 and 1031) and (986 and 1047) clustered significantly together in the subtype B clade and the latter pair reported close contact with each other (Figures 1 and 2). The intrapair HIV-1 *pol* nucleotide identity for both MSM pairs was >99.8%. Contact information was not available for the remaining two sequences (553 and 564) to investigate further their potential epidemiologic links. These three sets of B sequences still clustered together after phylogenetic analysis of over 4,000 global subtype B sequences in the GenBank database (Figure S3) further supporting their molecular and possible epidemiologic linkage.

### Association of major HIV-1 subtypes with demographic and risk groups in Bulgaria

Subtype B is the most frequent HIV-1 present in our study population and was found in approximately half (104/202, 51.5%) of the patient samples (Table 2, Figures 1 and 2). Nine patients (8.7%) reported acquiring HIV-1 outside of Bulgaria; one each in England, Greece, Italy, South Africa, Romania, Spain, two in Germany and one patient did not report a specific country where he was infected. In a multivariable model, controlling for age, gender, risk mode, country of infection, and calendar period of diagnosis, relative to other subtypes, subtype B was independently higher in MSM (odds ratio (OR)  = 16.0, p-value<0.001) and significantly lower in IDU (OR = 0.2, p-value = 0.02) compared to heterosexual risk, and significantly lower in females (OR = 0.3, p-value = 0.002) compared to males and persons ≥45 years of age at diagnosis (OR = 0.3, p-value 0.05) compared to persons 20–44 years of age (Table 3). There was a significant spike in the relative prevalence of subtype B during the 2000–05 period in our study population (OR = 3.5, p-value = 0.03). Phylogenetic analysis of over 4,000 global subtype B sequences showed that this subtype has been introduced into Bulgaria on multiple occasions followed by local expansion demonstrated by clustering of some Bulgarian sequences in this tree (Figure S3).

**Table 3:** Risk factors associated with major HIV-1 subtypes in Bulgaria, results from multivariable logistic regression model.

	B (n = 104)		C, A1, F1, H (n = 16)		01_AE (n = 40)		02_AG (n = 15)		04_cpx, 05_DF, 14_BG, 36_cpx, (n = 8)		URFs and sub-subtypes (n = 19)	
	OR^1^ (95% CL^2^)	*p^3^*	OR (95% CL)	*P*	OR (95% CL)	*p*	OR (95% CL)	*P*	OR (95% CL)	*p*	OR (95% CL)	*p*
**Age (years)**												
≤20	1.3 (0.4, 4.6)	0.63	2.0 (0.3, 12.1)	0.44	1.5 (0.4, 5.9)	0.55	1.7 (0.2, 19.1)	0.65	Ref		Ref	
20-44	Ref		Ref		Ref		Ref		Ref		Ref	
≥45	0.3 (0.1, 1.0)	0.05	9.6 (2.6, 35.0)	<0.001	0.5 (0.1, 2.5)	0.41	0.7 (0.1, 6.7)	0.77	0.7 (0.1, 8.4)	0.8	2.3 (0.6, 8.6)	0.23
**Gender**												
Male	Ref		Ref		Ref		Ref		Ref		Ref	
Female	0.3 (0.1, 0.6)	0.002	0.8 (0.2, 2.9)	0.71	2.5 (1.1, 5.4)	0.02	4.6 (1.2, 17.0)	0.02	5.6 (1.1, 27.9)	0.04	1.4 (0.5, 4.2)	0.49
**Route of infection**												
Heterosexual Other	Ref		Ref		Ref		Ref		Ref		Ref	
MSM	16.0 (3.3, 77.4)	<0.001	0.7 (0.1, 3.9)	0.69	Ref		Ref		Ref		Ref	
IDU	0.2 (0.1, 0.7)	0.02	Ref		4.0 (1.2, 13.0)	0.02	8.4 (2.0, 34.6)	0.003	1.6 (0.1, 21.8)	0.73	1.4 (0.1, 13.3)	0.78
**Presumed country of infection**												
Bulgaria	2.4 (0.8, 6.9)	0.10	2.5 (0.4, 14.5)	0.30	5.3 (0.6, 43.2)	0.12	0.6 (0.1, 4.8)	0.66	0.1 (0.0, 0.5)	0.006	0.2 (0.1, 0.6)	0.007
Abroad	Ref		Ref		Ref		Ref		Ref		Ref	
**Diagnosis period**												
1986-1995	Ref		Ref		Ref		Ref		Ref		Ref	
1996-1999	2.0 (0.5, 7.5)	0.31	0.3 (0.1, 2.2)	0.24	5.5 (0.6, 51.3)	0.14	0.5 (0.1, 12.0)	0.67	Ref		0.9 (0.2, 5.6)	0.95
2000-2005	3.5 (1.1, 11.2)	0.03	0.2 (0.1, 0.9)	0.03	4.5 (0.5, 38.0)	0.17	0.4 (0.1, 6.7)	0.56	0.1 (0.0, 1.5)	0.1	0.7 (0.2, 2.9)	0.59
2006-2009	2.3 (0.7, 8.1)	0.19	0.2 (0.1, 1.1)	0.06	2.3 (0.3, 20.8)	0.45	2.5 (0.2, 30.2)	0.48	0.7 (0.1, 4.1)	0.69	0.2 (0.1, 1.5)	0.13

1. Odds ratios (OR) are the estimated odds of a subtype occurring in the study population for a given risk factor relative to the referent group (Ref) as compared to occurrence of all other subtypes.

2. CL, confidence limit.

3. *p*, is the p-value for differences in subtype prevalence compared to the referent group.

HIV-1 subtype C was seen in seven (3.5%) persons (Table 2, Figures 1 and 3). Five (2.5%) persons were infected with subtype A1. Two persons (1.0%) were infected with subtype F1 (Table 2, Figures 1 and 4A) and their sequences clustered together with strong statistical support. Also, two persons (1.0%) were determined to be infected with subtype H (Table 1, Figures 1 and 3). Collectively, infections with non-B subtypes (C, F1, A1, and H) (n = 16, 7.9%) were more likely to occur among persons ≥45 years old (OR = 9.6, p-value <0.001) compared to persons 20-44 years old (Table 3).

### Association of circulating and complex recombinant HIV-1 forms with demographic and risk groups in Bulgaria

Phylogenetic and subtype analysis classified 63/202 (31.2%) HIV-1 sequences from Bulgaria as CRFs (Table 2, Figures 1, 5, 6A and B). CRF01_AE is the most common HIV-1 recombinant form in Bulgaria with 40 (19.8%) of the 202 sequences in our study. Based upon multivariable regression, compared to all other subtypes, CRF01_AE prevalence is significantly higher among females (OR = 2.5, p = 0.02) and IDUs (OR = 4.0, p = 0.02) relative to other risk groups combined (Table 3). Two of four newborns in the study were also infected with HIV-1 CRF01_AE reflective of more women with this subtype.

We found HIV-1 CRF02_AG to be the second most frequent CRF group in Bulgaria with 15/202 persons (7.4%) infected with this subtype (Table 2, Figures 1 and 5A). In a single-variable regression analysis (data not shown), compared to other subtypes, CRF02_AG infection was significantly higher during later diagnosis periods. However, this finding was explained by the coincidental increase in IDU infection over time. In the multivariate analysis, CRF02_AG prevalence was higher among IDU (OR = 8.4, p = 0.003) and females (OR = 4.6, p = 0.02) (Table 3) independent of other potential risk factors.

Four extremely rare HIV-1 genotypes were detected in Bulgaria among 8 persons (4.0%), including CRF04_cpx, CRF05_DF, CRF14_BG, CRF36_cpx, (Table 2, Figures 1, 6A and B). Only a limited number of CRF05_DF sequences have been reported worldwide [2]. Our sequence analyses identified four (1.98%) persons infected with HIV-1 CRF05_DF, two of whom reported traveling abroad.

Only one sequence (062) genotyped as HIV-1 CRF04_cpx and which clustered with strong support with similar reference strains from Cyprus, Greece and France (Table 2, Figures 1, 6A and B), countries where this complex form has only been identified to date [42]–[44]. Phylogenetic analysis of all CRF04_cpx sequences available at Los Alamos and GenBank comparable in size to our sequence confirmed the genotype of sequence 062 (Figure 6B).

One Bulgarian HIV-1 *pol* sequence (150) clustered with significant support with the reference CRF36_cpx sequence [2] (Table 2, Figures 1 and 6A). This patient reported probable acquisition of infection while in Libya but this rare subtype has only been found in Cameroon [2].

Two HIV-1 *pol* sequences (534 and 669) clustered with the referent CRF14_BG sequence (Table 2, Figures 1 and 6B). One of these patients (534) reported probable acquisition of infection while in Spain. CRF14_BG is widespread in Portugal and Spain, and in some African countries, and was also recently found among IDUs in Greece [45], [46]. Collectively, these rare HIV-1 genotypes were significantly less likely to be acquired in Bulgaria (OR = 0.1, p = 0.006) and were more likely to be found in women (OR = 5.6, p = 0.04) based upon multivariable analysis.

In addition to the 12 A and F sub-subtype variants described above, seven other persons were infected with novel URFs (Table 2, Figure 1, 6A and 6C). HIV-1 *pol* sequences from two patients (004 and 297) clustered together weakly but formed a unique lineage between subtypes C and BC with a strong posterior probability (Figure 1). Bootscan analysis revealed that the *pol* sequence from person 004, was composed of unidentified and subtype C sequences, whereas the HIV-1 *pol* from person 297, who reported possible infection abroad, was composed of subtype J and C sequences with strong support (Figure 6C and Figure S4A and B). Two sequences (021 and 452) clustered with genotype CRF34_01B phylogenetically but with weak bootstrap and posterior probability (Figure 1). Bootscan analysis showed sequence 021 was composed of A1, A2, and unidentified sequences, while sequence 452 was mostly a mixture of unidentified sequences (Figure 6C and Figure S4C and D). Sequence 063 formed a lineage ancestral to the HIV-1 A1 and CRF02_AG genotypes (Figures 1 and 6C) and was composed of *pol* sequences from A1 and undefined fragments (Figure S4E). Sequence 399 clustered with CRF45_cpx with weak support (Figure 1) but bootscan analysis revealed it was a complex of CRF45-cpx and CRF09-cpx sequences (Figure S4F). Sequence 316 clustered with CRF43_02AG reference sequences with strong posterior probability (Figures 1 and 6A), but was composed of both CRF43_02G and G fragments (Figure S4G). Based upon multivariable analysis, these URFs were significantly less likely to occur among persons infected while in Bulgaria (OR = 0.2, p = 0.0074) (Table 3).

### Trends in the HIV-1 subtype prevalence and total infections in high risk groups

**Figure 8:**
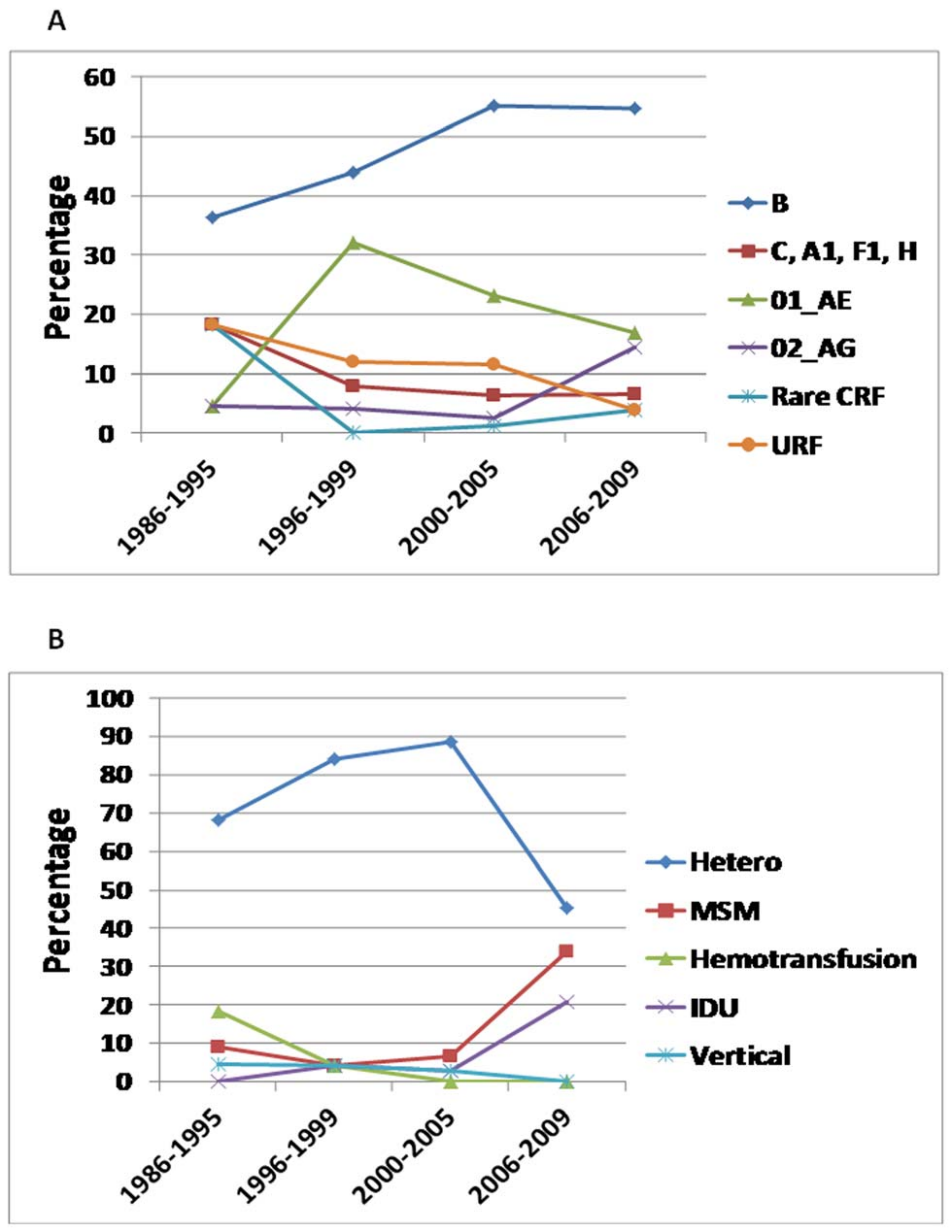
Trends in Bulgarian HIV-1 subtype (A) prevalence and (B) transmission route. HIV-1 genotypes were stratified into four time periods of the epidemic consisting of early (1986–1995), late 1990s (1996–1999), and the first (2000–2005) and second halves (2006–2009) of the last decade.

To better understand the temporal trends of the epidemiology of HIV-1 infection in Bulgaria and for purposes of targeting prevention resources, we analyzed trends in the prevalence of HIV-1 subtypes, and trends in the major risk groups across four time periods (1986–1995, 1996–1999, 2000–2005, and 2006–2009) without controlling for explanatory variables. The prevalence of subtype CRF02_AG increased from 4.5% in 1986–1995 to 14.3% in 2006-2009 (p = 0.04) despite the underrepresentation of IDUs in the study population and a strong positive association of this subtype with IDU (Table 2, Figures 8A and B). CRF01_AE prevalence decreased after 1996-1999 even though this subtype was also strongly associated with IDU and IDU prevalence increased during the last time period (Table 3, Figures 8A and B). The overall CRF02_AG and CRF01_AE prevalences decreased during the last period because the larger proportion of heterosexual infections in our study (140/202) with these subtypes decreased during the last period which was greater than the number of new IDU infections with these CRFs. Subtype B did not significantly increase over time using multivariable or single variable analyses (data not shown) despite a significant spike in prevalence from 2000–2005 (OR = 3.5, p-value = 0.03) likely explained by overrepresentation of MSM in the study population and a simultaneous decrease in infected heterosexuals during that period (Figures 8A and B). The rare subtypes 04_cpx, 05_DF, 14_BG, 36_cpx, were most prevalent during 1986-1999 (18.2%), but have declined to 3.9% in 2006-2009 (p = 0.03).

Proportions of younger infected persons, ages <20, were significantly declining (trend test p<0.001). Persons infected through heterosexual contact and vertical transmission were declining, while those infected through MSM or IDU were increasing (p<0.001) (Figure 8B). During the early years of the epidemic, HIV-1 infection was greatest among Bulgarian citizens returning from abroad. However, there was an increasing trend in the proportion of persons infected in Bulgaria (p = 0.02), particularly during 2006-2009 when 92.2% acquired infection within the country.

## Discussion

In the present study we describe the expanded molecular surveillance and epidemiological assessment of the HIV-1 epidemic in Bulgaria. Our results extend those reported previously by our group that initially identified multiple subtypes in Bulgaria, which we proposed were sustained by viral inflow from European countries, USA and Africa [27]. In addition to our earlier results, current national data from ongoing HIV and AIDS surveillance suggest that the HIV/AIDS incidence in Bulgaria remains low overall. To obtain a broader picture of the HIV-1 epidemic in Bulgaria we analyzed new *pol* sequences from 125 HIV-1-infected persons and combined these with 77 *pol* sequences from patients reported in our previous study, altogether representing about 20% of infections identified to date. Our study shows that the HIV-1 epidemic in Bulgaria has several key characteristics relative to the genetic composition of circulating viruses in the country, including a high genetic diversity of more than 15 different HIV-1 subtypes, CRFs and URFs, two new sub-subtype variants, and the introduction of seven extremely rare HIV-1 forms, including CRF05_DF, CRF04_cpx CRF36_cpx, CRF14_BG, and a plethora of different URFs that are distinct from all reference sequences. The data suggest the presence of local networks in certain risk groups with specific HIV-1 genotypes [29], an unequal subtype distribution among different risk groups, with most MSM infected with subtype B, and the lack or low prevalence of certain genotypes in specific groups, and the fluctuation of different HIV-1 genotypes overtime and in certain risk groups.

In our current study we found a broader genetic heterogeneity of HIV-1 circulating in Bulgaria than that previously reported by our group [27]. Most infections were with the major HIV-1 subtypes followed by a variety of CRFs, URFs and sub-subtypes. Although subtype B was the most prevalent genotype in both studies and represents about half the HIV-1 infections in Bulgaria, our results differ from those in Western Europe where subtype B comprises the vast majority of infections [2], [47], [48]. Our results show that subtype B has been introduced into Bulgaria multiple times, but also with local expansion in certain populations. In contrast to our previous study [27], [48], the second largest genotype in Bulgaria is now CRF01_AE, followed by CRF02_AG. We also identified seven new complex circulating forms not seen before in Bulgaria and which are typically found in Cameroon, Libya, Singapore, Thailand, Saudi Arabia, Greece, Cyprus, Portugal, Spain, or France. Notably, two of these CRFs have been associated with outbreaks in IDUs in Greece (CRF04_cpx and CRF14_BG) [43]–[46], but only one of these genotypes (CRF14_BG) was present in an IDU in Bulgaria and may represent a recent infection. The others were present in non-IDU populations (three heterosexual and one blood product recipient) suggesting that these rare variants may be spreading outside of IDUs. We also identified nine possible new URFs circulating in Bulgaria that may be the result of the mixing of so many genotypes in the population similar to the natural history of HIV-1 in Africa. These URFs were found to be associated with persons reporting travel abroad who may be facilitating the increase of viral diversity in Bulgaria. All of the URFs identified in our study, including the clusters of A-like and F-like sequences, will require further characterization using complete genomes to determine their genetic composition and final classification as was done for subtype K and the A and F sub-subtypes [41], [49]–[51].

The distribution of HIV-1 subtypes in our study population varied by age, sex, geography, and risk exposure. The greatest number of infections and broadest HIV-1 diversity occurred in major cities where most immigrants tend to live. We also observed the largest number of HIV subtypes and hence the greatest amount of HIV diversity in persons over 45 years old despite their being fewer total infections in this age group. Persons infected by heterosexual or IDU transmission had the greatest variety of subtypes, compared to MSM (p<0.0001). Furthermore, we also observed that closely related viral clades are increasing in circulation in geographically restricted IDU subgroups. The HIV-1 CRF02_AG seen in one IDU subgroup appears to have been transmitted within separate transmission clusters. Both women and IDUs were more likely to be infected with subtypes CRF01_AE and CRF02_AG, though the trend in IDUs may be due to an overall increase in IDU infections over time in Bulgaria. In contrast, with the exception of two persons, all MSM were infected with subtype B. Our results are consistent with several studies from different European countries that found a predominance of subtype B in MSM [22], [47], [48], [52]. There is also recent independent evidence that subtype B in Bulgaria may be phylogenetically related to subtype B strains identified in Russia, Greece and some other neighboring Balkan countries [47]. In addition, our phylogenetic and epidemiologic analyses showed an absence or reduced prevalence of certain viral strains in specific populations. For example, neither MSM nor individuals infected by blood transfusion were infected with HIV-1 CRF01_AE, which was the second most common genotype in Bulgaria. These findings suggest a pattern of independent and limited transmission of some viral clades within certain population groups without viral exchange between them. The high level of HIV-1 diversity and wide distribution among various risk groups and demographic classifications are likely to influence various aspects of the epidemic in Bulgaria, including patient treatment, disease progression, laboratory monitoring, and vaccine development.

Analysis of longitudinal epidemiologic and demographic data with HIV-1 genotype can provide important information regarding infection dynamics during different stages of the epidemic in Bulgaria. Using this strategy by year of HIV/AIDS diagnosis we found a dissimilar rate of introduction and spread of different HIV-1 clades over time during the history of the epidemic in Bulgaria. Some clades persist, such as subtype B, while others like CRF01_AE and subtype C have declined, and other clades appear to be emerging, like CRF02_AG. We also found that there was a significant increase in the number of infections in MSM and IDUs with a concomitant decline in heterosexual and vertical infections. Some of these observed changes in the dynamics of HIV-1 infection in Bulgaria can be explained by the initial introduction of specific HIV-1 clades in certain populations spreading afterwards within populations at increased risk for infection. We also found that during the early years of the epidemic HIV-1 was most likely found in Bulgarians returning from abroad and who then transmitted HIV-1 via heterosexual contact. However, in recent years the significant increase in transmission of certain HIV-1 subtypes to and among IDUs and MSM is most likely due to increased risky behavior and spread of subtypes within these groups but also coincided with a decrease in heterosexual infections.

Our results also suggest that the infection dynamics and HIV-1 genetic diversity of the HIV-1 epidemic in Bulgaria are fluctuating. There are a few factors that seem relevant to help explain this phenomenon. Firstly, the central geographic location of Bulgaria at the crossing point between Western Europe, Eastern Europe, and the Middle East facilitates the introduction of HIV-1 into and beyond Bulgaria. In addition, an increase of West African immigrants to Europe to escape war, oppression and poverty has likely helped increase the HIV-1 diversity in the country [53]. The spread of HIV-1 within high risk groups like IDUs and MSM from 2006–2009 supports the changing epidemiological dynamics of HIV-1 infection which could indicate an increase of these populations in Bulgaria or increases in risky behavior or both. There has been a dramatic reduction and inversion of the socio-economic status of the Bulgarian population from a planned to a market economy after 1989 associated with high unemployment rates of about 20% in 2001 prior to the period of our study and a subsequent fall in the gross domestic product by 50%, which may lead individuals to substance abuse with an increased risk for infection (www.google.com/publicdata). An underestimation of MSM transmission in the early years of the epidemic due to stigma associated with reporting of homosexual contacts could also explain the observed increase in MSM infections. Thus, control of the HIV-1 epidemic in these populations will require targeted interventions in conjunction with continuous and increased surveillance efforts of specific at-risk groups, especially IDUs and MSM and their contacts. These public health initiatives will be crucial to understanding the details of the epidemiology of HIV-1 in Bulgaria and to assess the efficacy of prevention strategies and control of the epidemic.

The cross-sectional design of our study, which included convenience specimens, collected as patients were diagnosed or came into clinics for care, may not truly represent the other 80% of reported cases in Bulgaria. The effect of this possible sampling error in our analyses is unknown but may influence genotype distribution over time and in different populations. For example, we determined that MSM were overrepresented and IDUs were underrepresented in our two studies combined which may affect the trend interpretations in these risk groups. Increased HIV-1 testing and surveillance, especially in IDUs, should help to overcome this potential bias and improve the accuracy of understanding the epidemic in Bulgaria and the proposed targeted interventions. Also, our phylogenetic results are based on a single genomic region, in some cases genotypes were only identified from limited numbers of patients. Thus, confirmation of the rare genotypes identified herein may require sequence analysis of additional regions or complete genomes and their detection in larger numbers of persons. Any potential association of infection with a geographic location is based on self-reporting and may be affected by recall or other biases and thus may require analysis of larger HIV-1 datasets from the purported country where the transmission was reported to confirm the origin. Minor differences between our results and those of our previous study most likely represent the use of more stringent criteria for genotype classification and the use of a larger set of reference sequences that included more CRFs in the current study.

In conclusion, we found a broad level of HIV-1 genetic diversity in Bulgaria, including most major subtypes and CRFs and the identification of novel URFs and sub-subtypes, whose composition fluctuates in different populations and during different phases of the epidemic. Our data also suggest the extremely dynamic nature of the Bulgarian epidemic is characterized by an unequal distribution of different HIV-1 genotypes among high risk populations. These findings emphasize the need for sustained and focused molecular epidemiological surveillance to identify transmission links that can be targeted by prevention strategies to control the HIV-1 epidemic in Bulgaria.

## Supporting Information

Figure S1 **Subtype and genetic composition of HIV-1 F-like sub-subtypes in Bulgaria.** Subtype and genetic composition of protease-polymerase sequences was inferred using manual bootscan analysis with the program SimPlot using the maximum likelihood (F84) nucleotide substitution model, a sliding window of 200-bp, a 40-bp step, with the transition/transversion ratio determined empirically. The standard recombination cutoff of 70% of permuted trees is indicated with a red dashed line. (A) query sequence 133; (B) query sequence 207; (C) query sequence 210; (D) query sequence 228; (E) query sequence 376; (F) query sequence 597; (G) query sequence 906.
(TIF)

Figure S2 **Subtype and genetic composition of HIV-1 A-like sub-subtypes in Bulgaria.** Subtype and genetic composition of protease-polymerase sequences was inferred using manual bootscan analysis with the program SimPlot using the maximum likelihood (F84) nucleotide substitution model, a sliding window of 200-bp, a 40-bp step, with the transition/transversion ratio determined empirically. The standard recombination cutoff of 70% of permuted trees is indicated with a red dashed line. (A) query sequence 434; (B) query sequence 540; (C) query sequence 544; (D) query sequence 584; (E) query sequence 833.
(TIF)

Figure S3 **Global phylogenetic relationships of Bulgarian HIV-1 subtype B polymerase sequences.** The 753-bp alignment consisted of >4,000 reference sequences sampled globally and 104 subtype B sequences from Bulgaria. Phylogenetic tree was inferred by the maximum likelihood method as implemented in the FastTree program (http://www.microbesonline.org/fasttree/) using a general time reversible (GTR) model with 20 gamma categories. The reliability of the clusters was assessed with the Shimodaira-Hasegawa test implemented in the FastTree program. Red diamonds indicate Bulgarian sequences.
(TIF)

Figure S4 **Subtype and genetic composition of HIV-1 unique recombinant forms (URFs) in Bulgaria.** Subtype and genetic composition of protease-polymerase sequences was inferred using manual bootscan analysis with the program SimPlot using the maximum likelihood (F84) nucleotide substitution model, a sliding window of 200-bp, a 40-bp step, with the transition/transversion ratio determined empirically. The standard recombination cutoff of 70% of permuted trees is indicated with a red dashed line. (A) URF query sequence 004; (B) URF query sequence 297; (C) URF query sequence 021; (D) URF query sequence 452; (E) URF query sequence 063; (F) URF query sequence 399; (G) URF query sequence 316.
(TIF)
