# Supplementary figures and images for: Detailed Molecular Epidemiologic Characterization of HIV-1 Infection in Bulgaria Reveals Broad Diversity and Evolving Phylodynamics
Source: PLoS One. 2013 Mar 19;8(3):e59666. doi: 10.1371/journal.pone.0059666 (PMC3602066; doi:10.1371/journal.pone.0059666)

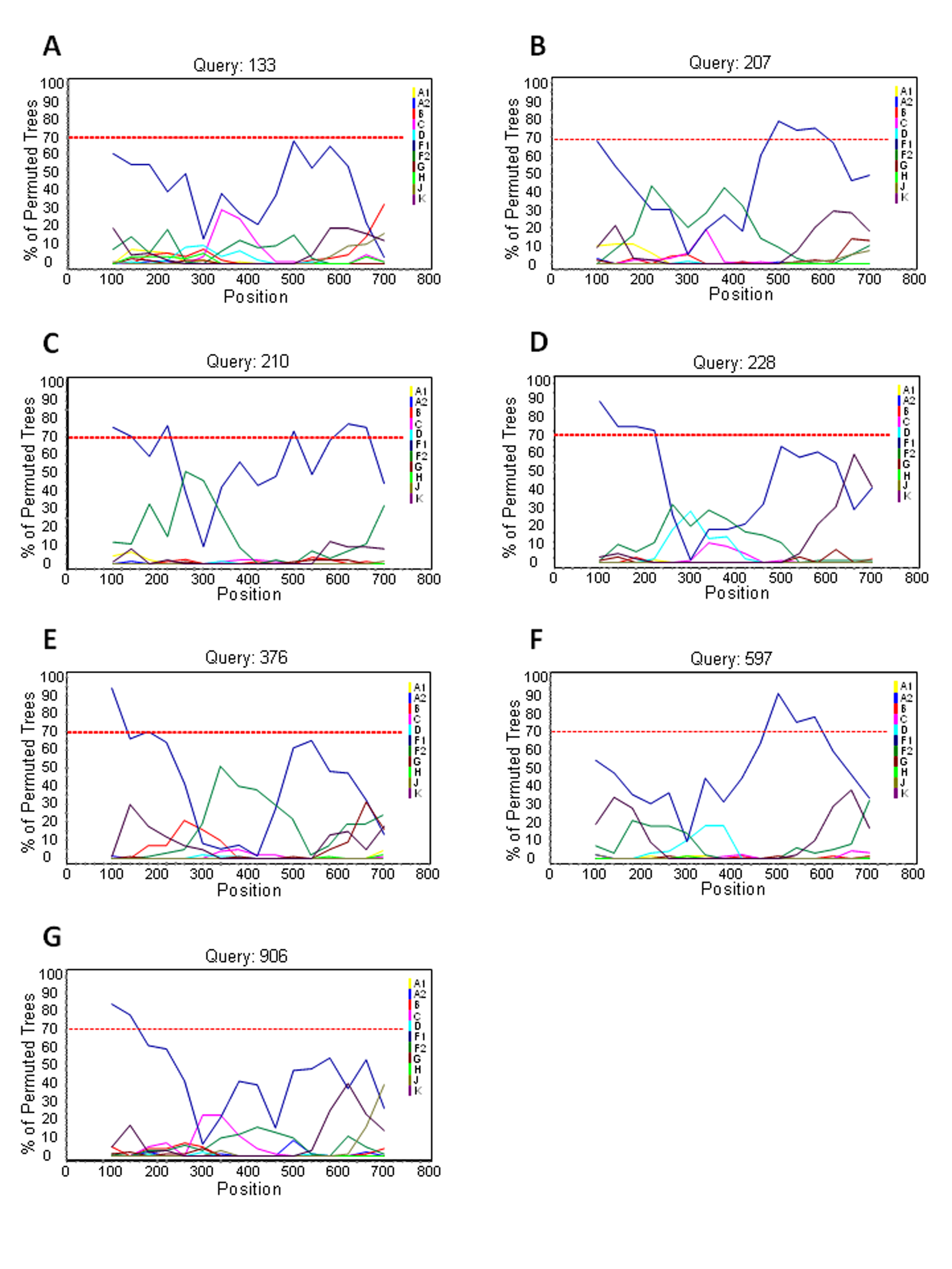

Supplement: Figure S1 — Subtype and genetic composition of HIV-1 F-like sub-subtypes in Bulgaria. Subtype and genetic composition of protease-polymerase sequences was inferred using manual bootscan analysis with the program SimPlot using the maximum likelihood (F84) nucleotide substitution model, a sliding window of 200-bp, a 40-bp step, with the transition/transversion ratio determined empirically. The standard recombination cutoff of 70% of permuted trees is indicated with a red dashed line. (A) query sequence 133; (B) query sequence 207; (C) query sequence 210; (D) query sequence 228; (E) query sequence 376; (F) query sequence 597; (G) query sequence 906. (TIF) [file pone.0059666.s001.tif]

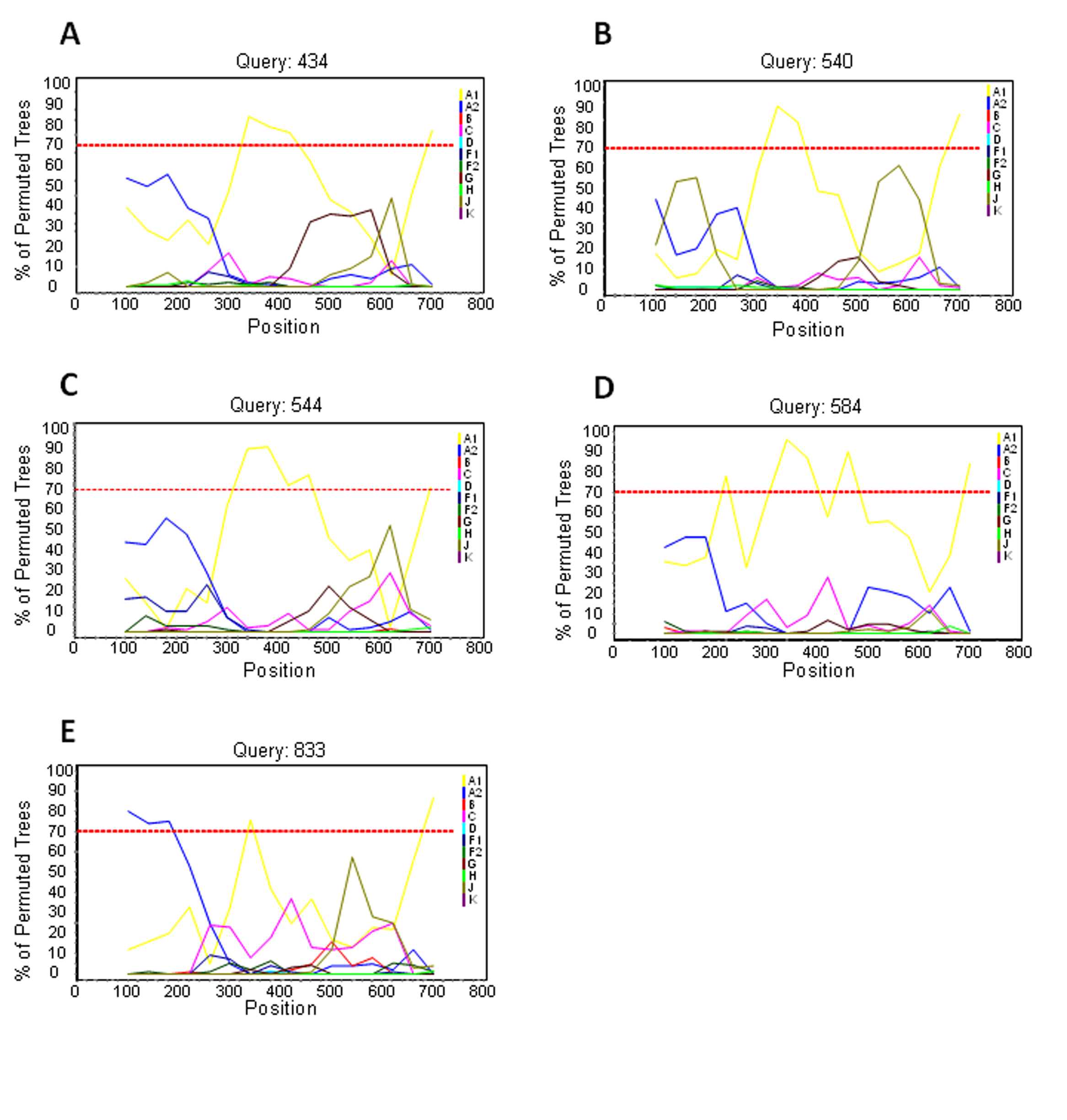

Supplement: Figure S2 — Subtype and genetic composition of HIV-1 A-like sub-subtypes in Bulgaria. Subtype and genetic composition of protease-polymerase sequences was inferred using manual bootscan analysis with the program SimPlot using the maximum likelihood (F84) nucleotide substitution model, a sliding window of 200-bp, a 40-bp step, with the transition/transversion ratio determined empirically. The standard recombination cutoff of 70% of permuted trees is indicated with a red dashed line. (A) query sequence 434; (B) query sequence 540; (C) query sequence 544; (D) query sequence 584; (E) query sequence 833. (TIF) [file pone.0059666.s002.tif]

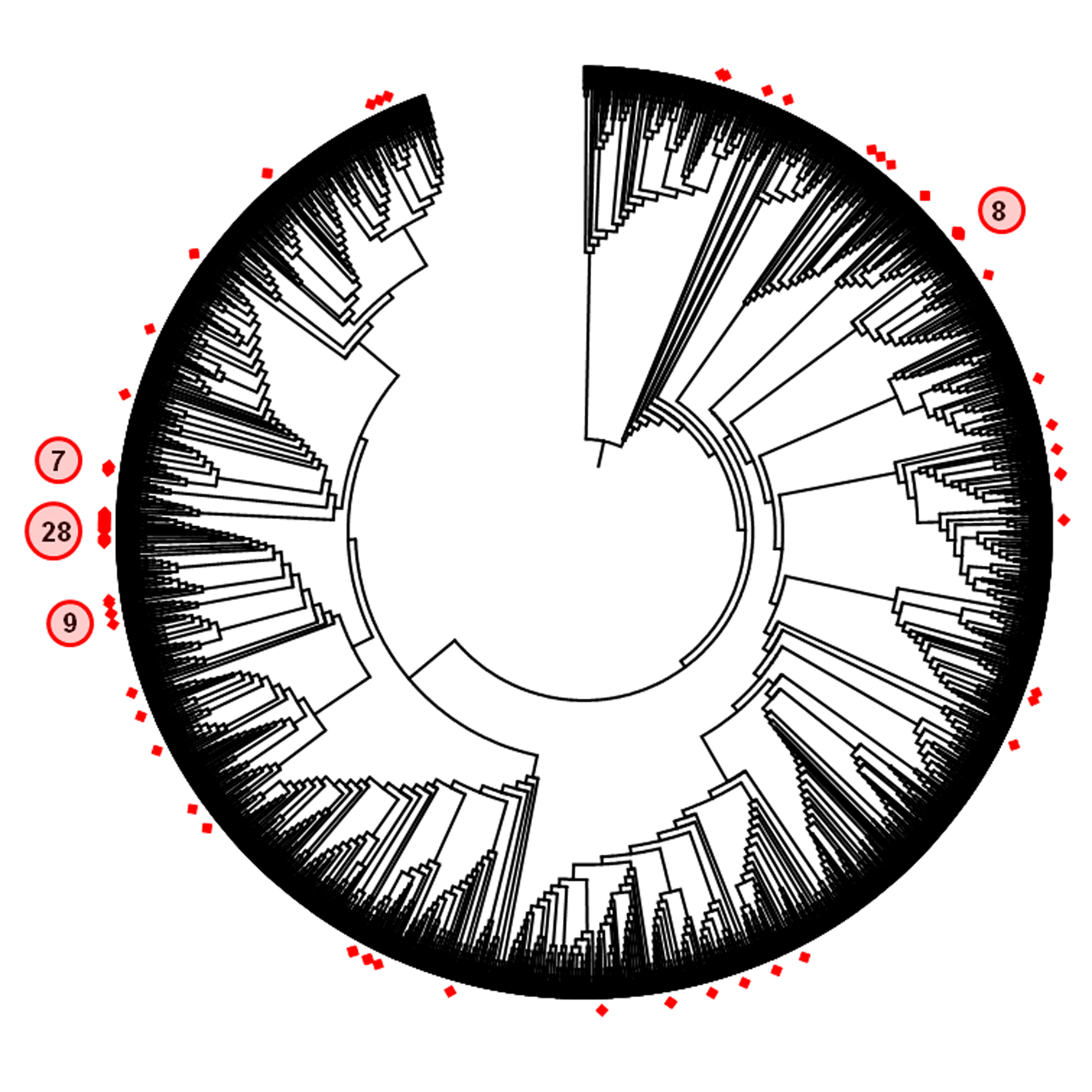

Supplement: Figure S3 — Global phylogenetic relationships of Bulgarian HIV-1 subtype B polymerase sequences. The 753-bp alignment consisted of >4,000 reference sequences sampled globally and 104 subtype B sequences from Bulgaria. Phylogenetic tree was inferred by the maximum likelihood method as implemented in the FastTree program (http://www.microbesonline.org/fasttree/) using a general time reversible (GTR) model with 20 gamma categories. The reliability of the clusters was assessed with the Shimodaira-Hasegawa test implemented in the FastTree program. Red diamonds indicate Bulgarian sequences. (TIF) [file pone.0059666.s003.tif]

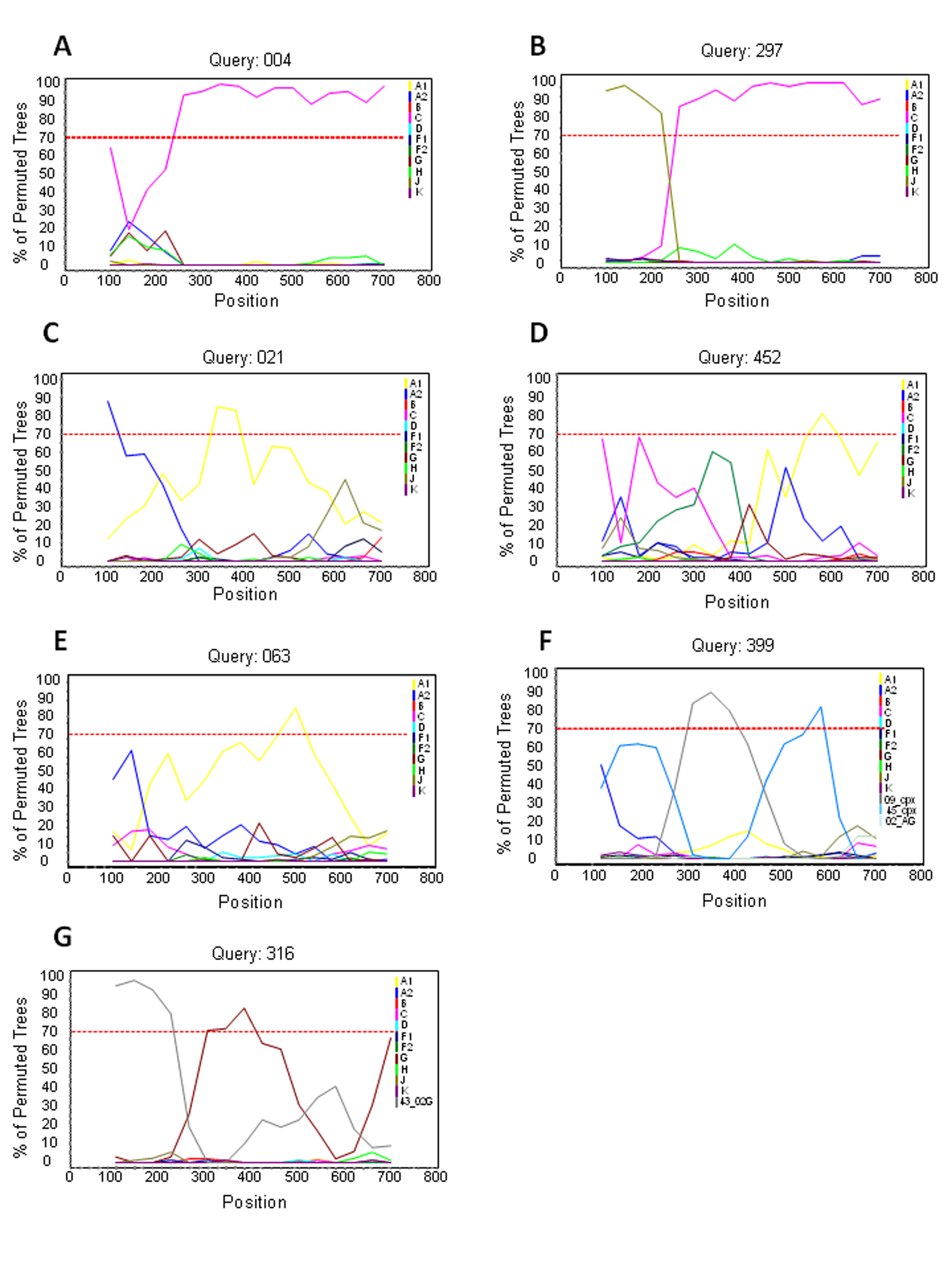

Supplement: Figure S4 — Subtype and genetic composition of HIV-1 unique recombinant forms (URFs) in Bulgaria. Subtype and genetic composition of protease-polymerase sequences was inferred using manual bootscan analysis with the program SimPlot using the maximum likelihood (F84) nucleotide substitution model, a sliding window of 200-bp, a 40-bp step, with the transition/transversion ratio determined empirically. The standard recombination cutoff of 70% of permuted trees is indicated with a red dashed line. (A) URF query sequence 004; (B) URF query sequence 297; (C) URF query sequence 021; (D) URF query sequence 452; (E) URF query sequence 063; (F) URF query sequence 399; (G) URF query sequence 316. (TIF) [file pone.0059666.s004.tif]
